# Development of Diagnostic Markers and Applied for Genetic Diversity Study and Population Structure of *Bipolaris sorokiniana* Associated with Leaf Blight Complex of Wheat

**DOI:** 10.3390/jof9020153

**Published:** 2023-01-23

**Authors:** Abhijeet Shankar Kashyap, Nazia Manzar, Avantika Maurya, Deendayal Das Mishra, Ravinder Pal Singh, Pawan Kumar Sharma

**Affiliations:** 1 Molecular Biology Lab, ICAR-National Bureau of Agriculturally Important Microorganisms, Maunath Bhanjan 275103, India; 2 Plant Pathology Lab, ICAR-National Bureau of Agriculturally Important Microorganisms, Maunath Bhanjan 275103, India; 3 ICAR-Indian Agricultural Research Institute, New Delhi 110012, India; 4 Department of Microbiology, Dr. R.M.L Avadh University, Faizabad 224001, India

**Keywords:** microsatellite markers, polymorphism information content, leaf blight of wheat, SSR

## Abstract

*Bipolaris sorokiniana*, a key pathogenic fungus in the wheat leaf blight complex, was the subject of research that resulted in the development of fifty-five polymorphic microsatellite markers. These markers were then used to examine genetic diversity and population structure in Indian geographical regions. The simple sequence repeat (SSR) like trinucleotides, dinucleotides, and tetranucleotides accounted for 43.37% (1256), 23.86% (691), and 16.54% (479) of the 2896 microsatellite repeats, respectively. There were 109 alleles produced by these loci overall, averaging 2.36 alleles per microsatellite marker. The average polymorphism information content value was 0.3451, with values ranging from 0.1319 to 0.5932. The loci’s Shannon diversity varied from 0.2712 to 1.2415. These 36 isolates were divided into two main groups using population structure analysis and unweighted neighbour joining. The groupings were not based on where the isolates came from geographically. Only 7% of the overall variation was found to be between populations, according to an analysis of molecular variance. The high amount of gene flow estimate (N_M_ = 3.261 per generation) among populations demonstrated low genetic differentiation in the entire populations (F_ST_ = 0.071). The findings indicate that genetic diversity is often minimal. In order to examine the genetic diversity and population structure of the *B. sorokiniana* populations, the recently produced microsatellite markers will be helpful. This study’s findings may serve as a foundation for developing improved management plans for the leaf blight complex and spot blotch of wheat diseases in India.

## 1. Introduction

Wheat production during the green revolution contributed to the achievement of food security in different regions of the world with the highest population density [1]. The world’s population is expanding quickly, particularly in developing nations such as India, Pakistan, Bangladesh, and Nepal, and thus the demand for wheat production is increasing [2]. We need to produce between 840 and 1050 million tonnes of wheat to meet the increasing population’s demand in 2022 AD [3]. Past mistakes have taught us that there are many obstacles to successful wheat production for achieving the goal of feeding the world’s expanding population in the years to come. The incidence of biotic-stress-related diseases is one of the major factors reducing wheat harvest [4]. Among these diseases, the leaf blotch or foliar blight caused by *Bipolaris sorokiniana* (Sacc. in Sorok) in warm, humid areas of South Asian nations, Shoem, also known as *Helminthosporium sativum*, teleomorph (*Cochliobolus sativa*), is one of the most destructive pathogens [5,6]. Small, dark brown lesions that eventually grow into light to dark brown oval or elongated blotches are the first signs of infection. These elongated spots eventually unite to form necrotic lesions, which cause significant tissue loss as the disease progresses. According to reports, yield losses in sensitive genotypes might range from 15.5 to 19.6% and can reach 100% under severe conditions [7,8]. Spot blotch infection causes black, undersized, discoloured, and shrivelled pointed seeds that have an impact on grain quality and trade. Both domesticated and wild Poaceae species have been proven to be susceptible to the fungus [9]. *B. sorokiniana* has a broad host range, causing the pathogen to have a high genetic heterogeneity [10,11]. The significant genetic variation of the pathogens and the role of minor genes in determining resistance to foliar blight or spot blotch present breeders and plant pathologists with ongoing challenges. It is critical to comprehend the *B. sorokiniana* fungus’ genetic variety by molecular characterisation. The variety of *B. sorokiniana* isolates identified in wheat-producing regions has to be studied [12,13,14,15]. The ecology is also impacted by these approaches, which unfortunately take a lot of time and work. Their accuracy and precision are also very lacking. DNA-based markers, on the other hand, have been demonstrated to be precise and accurate and to be able to comprehend the genetic variation of fungi diseases [16,17,18]. Numerous molecular markers, including SNPs, ISSRs, AFLPs, and RAPD, have been used to examine the genetic diversity and population structure of fungal crop pathogens [19,20,21,22,23]. Due to SSR polymorphic nature, great reproducibility, co-dominance, and universality within the genome, SSR has emerged as a viable marker for genotyping, linkage mapping, detecting QTL, genome analysis, plant breeding, and evolutionary studies [24,25,26,27].

The current work focused on the keypathogen of the leaf blight complex, *B. sorokiniana* isolates, and the establishment of SSR markers, genetic diversity, and population structure in Indian geographic conditions. *Bipolaris sorokiniana* is characterised by a lack of physiologic specialisation in the pathogen and a continuous change in symptom expression that is quantitative rather than qualitative. This study set out to detect and evaluate polymorphic SSR markers, pathogen genetic diversity, and other factors affecting the population structure of *B. sorokiniana*.

## 2. Materials and Methods

### 2.1. Fungal Isolate Collection

**Figure 1:**
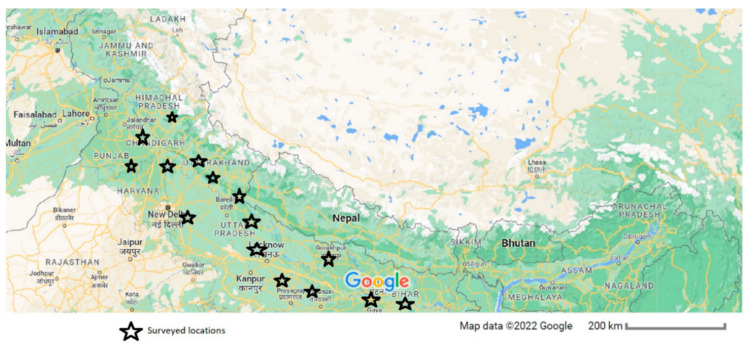
A map showing surveyed area for the collection of leaf blight samples of wheat in India.

The Indo-Gangetic plains of India were the source of 187 collected samples of wheat with leaf blight symptoms (Figure 1, Table S1). In winter 2016–2017, 106 samples were collected, while 81 samples were collected the following year. The fungi were isolated from around 60% of the symptomatic leaf tissue samples obtained from 17 sites in India. The isolates were subcultured on potato dextrose agar (PDA) medium amended with streptomycin sulphate (125 ppm) and incubated at 26 °C with a 12 h light period, being later stored at 4 °C. Further, these isolates were characterised at the molecular level and identified as *Bipolaris sorokiniana.* The GenBank IDs were MZ489399; MZ489401; MZ489402; MZ489406; MZ489414; MK676000; MK676001; OQ225200; OQ225201; OQ225202; OQ225203; OQ225204; OQ225205; OQ225206; OQ225207; OQ225208; OQ225209; OQ225210; OQ225211; OQ225212; OQ225213; OQ225214; OQ225215; OQ225216; OQ225217; OQ225218; OQ225219; OQ225220; OQ225221; OQ225222; OQ225223; OQ225224; OQ225225; OQ225226; OQ225227; and OQ225228.

### 2.2. Fungi Isolation and DNA Extraction

The total genomic DNA was extracted from a 14-day-old culture grown on PDA using the DNeasy Plant Mini Kit (Qiagen, Valencia, CA, USA) according to the manufacturer’s instructions. DNA concentrations were calculated using a nanodrop spectrophotometer. Prior to usage, DNA was kept at −20 °C. DNA was regularly diluted to 1:10 (*v*/*v*) in Tris EDTA buffer (PCR) before performing a polymerase chain reaction.

### 2.3. Microsatellite Development and Bioinformatics

The whole genome sequences of *B. sorokiniana* (PRJNA53923) available in the NCBI database were screened for SSR motifs (Supplementary Materials). With default settings, the Perl script MISA [28] was used to determine the relative abundance and frequency of repeating motifs. For the PCR amplification, fifty-five SSR primers that are present in the genome were chosen at random. PRIMER3 online software was used to develop primers [29].

### 2.4. Microsatellite PCR Amplification and Genotyping

The ideal annealing temperature for the PCR amplification of each SSR locus was determined using standard gradient PCR. The PCR assay was optimised in a final volume of 10 µL containing GoTaq Green Master Mix (Promega), 0.5 pmol of each forward and reverse primer, and 50 ng of fungal DNA. The cycling parameters are for initial denaturation at 94 °C for 4 mins, followed by 35 cycles of denaturation at 94 °C for 60 s, annealing at temperatures corresponding to each primer pair as mentioned in Table 1 for 1 min extension at 72 °C, and a final extension at 72 °C for 5 min. To reveal polymorphisms and for allele identification, the PCR products were analysed on 3 % (*w*/*v*) agarose gels stained with ethidium bromide and exposed to UV light to visualise DNA fragments. A 100 bp DNA ladder (Promoga) was used to estimate the amplicon sizes. The experimental isolates’ SSR markers were graded on the basis of whether or not the appropriate bands were present (Figure S1) [30].

### 2.5. SSR Polymorphism and Genetic Diversity

The gels were graded according to whether or not they contained pronounced, repeatable amplicons. Every amplicon was given the designation of a locus with two possible alleles. The SSR amplification data from several isolates were converted into discrete variables in a binary data matrix (0 = absence and 1 = presence). To explore the variation in partitioning between populations, a cluster analysis using the Unweighted Pair Group Method with Arithmetic Mean (UPGMA) algorithm was carried out using the NTSYS version 2.1 programme [31]. Using SSR markers, the genetic diversity of 36 *B. sorokiniana* isolates from various agroecological zones in India was examined. GenAlEx version 6.503 [32] was used to estimate the basic statistics, such as major allele frequency, the number of alleles per locus, heterozygosity, polymorphic information content (PIC), and gene diversity. The PIC value for each SSR marker was estimated using the formula as described by Kashyap et al. (2022) [33]. Additionally, Shannon’s information index, observed heterozygosity, expected heterozygosity, observed heterozygosity, number of private alleles per locus, banding pattern across the population, and number of effective alleles per locus were estimated for each population.

### 2.6. Population Structure and Gene Flow

The cumulative allelic diversity (Ht), mean allelic diversity within populations (Hs), percentage of total allelic diversity (Gst), and gene flow (Nm) within populations were all calculated using POPGENE version 1.31 software [34]. Using the computer application GenAlEx 6.5, the hierarchical analysis of molecular variance (AMOVA) was carried out. STRUCTURE 2.3.4 was used to analyse the population structure (Pritchard et al., 2000). Testing *K* = 1 to *K* = 15 with five different runs of 25,000 burn in period length at fixed iterations of 100,000 allowed for the most advantageous number of populations (*K*) to be determined. The optimum *K*-value was standardised by following the methodology of [35].

## 3. Results

### 3.1. Detection and Distribution of SSRs in B. sorokiniana Genome

**Figure 2:**
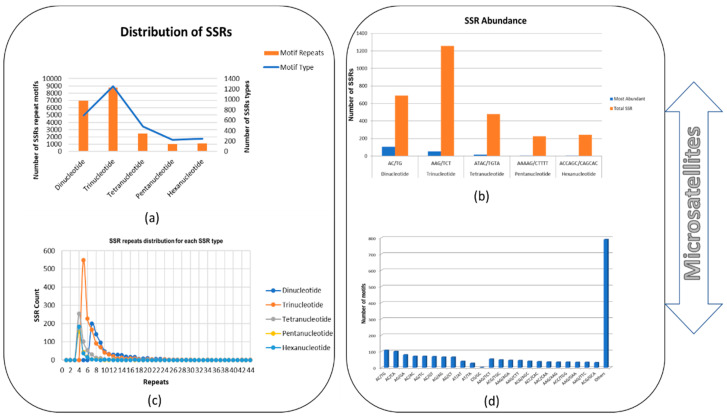
Showing distribution of SSRs in *B. sorokiniana* genome: (**a**) number of SSR repeat motifs; (**b**) SSR abundance; (**c**) SSR repeat distribution; (**d**) frequency of dinucleotide and trinucleotide SSR repeat motifs in the genome.

A total of 3251 SSR loci were discovered as an outcome of the genome sequence search for *B*. *sorokiniana*. The most frequent repeat motifs corresponding to dinucleotide to hexanucleotide repeats were (AC/TG)n, (AAG/TCT)n, (ATAC/TGTA)n, (AAAAG/CTTTT)n, and (ACCAGC/CAGCAC)n. The SSR repeat types varied—trinucleotides, dinucleotides, and tetranucleotides, for instance, accounted for 43.37% (1256), 23.86% (691), and 16.54% (479) of the 2896 SSR repeats, respectively. Pentanucleotide and hexanucleotide repeat motifs took up the remaining space, contributing to 7.80% (226) and 8.42% (244), respectively. When all SSR repeat motifs were considered, it was discovered that trinucleotide repeats made up the bulk of the motifs, while pentanucleotide repeats made up the least amount of them, as shown in Figure 2a,c.

Along the complete *B. sorokiniana* genome, the frequency of each SSR motif type was also determined. The most frequent dinucleotide motif was AC/TG (15.48%), followed by AC/CA (14.47%), AG/GA (11.43%), and AC/AC (10.13). The CG/GC pattern repetitions, meanwhile, were infrequently seen (0.05%). The trinucleotide predominant motif repeats were AAG/TCT (4.21%), ACG/TGA (3.82%), AAG/AGA (3.58%), and AAG/CTT (3.50%), and cumulatively the other 789 trinucleotides motifs shared 62.81% of dominant SSRs. Additionally, ATAC/TACA (3.34%) were the most frequently discovered tetranucleotide repetitions (Figure 2b,d).

### 3.2. Polymorphism of SSR Markers

The outcomes of gel electrophoresis on a number of highly polymorphic SSR markers are displayed in Figure S1. With the abovementioned amplified SSR primers, a total of 109 alleles were discovered. Each of these markers had an average of 2.36 alleles (Na) per locus. With an observed average of 1.6956 alleles, the effective number of alleles (Ne) per locus ranged from 1 to 2.8364. The average major allele frequency (MAF) was 0.6805, with a low number of 0.4737 and a high number of 0.9231. The observed heterozygosity (Ho), meanwhile, varied up to 0.8523. It was also revealed that the expected heterozygosity (He), with an average of 0.5013, ranged from 0.158 to 0.8563. Additionally, the polymorphic information content (PIC) ranged from 0.1319 to 0.5932, with a mean value of 0.3451, and the Shannon information index (I) varied from 0.2712 to 1.2415, with a mean value of 0.6509. The mean gene diversity in the current study was determined to be 0.4019. It was discovered that the various markers showed various polymorphisms. The most informative marker was found to be SSR24 (with PIC value: 0.5932), and the least informative marker was SSR49 (PIC value: 0.1319). This study came to the conclusion that when taken as a whole, the performances of the chosen SSR markers were very good at detecting genetic variation (Table 2).

### 3.3. Analysis of Molecular Variance

AMOVA’s outcome (Table S1) showed that *B. sorokiniana* isolates had a high genetic diversity (90%), but that genetic diversity among populations was minimal (3%). Very small but significant genetic distance values between population 1 (Hills) and population 2 (Plains) were revealed in a pairwise analysis (*p* < 0.001). Between the populations of the Hill and Plain, an average and consistent level of gene flow (Nm = 3.261) was observed, and a pairwise study revealed a genetic identity of 0.071 levels.

### 3.4. Population Genetic Structure

**Figure 3:**
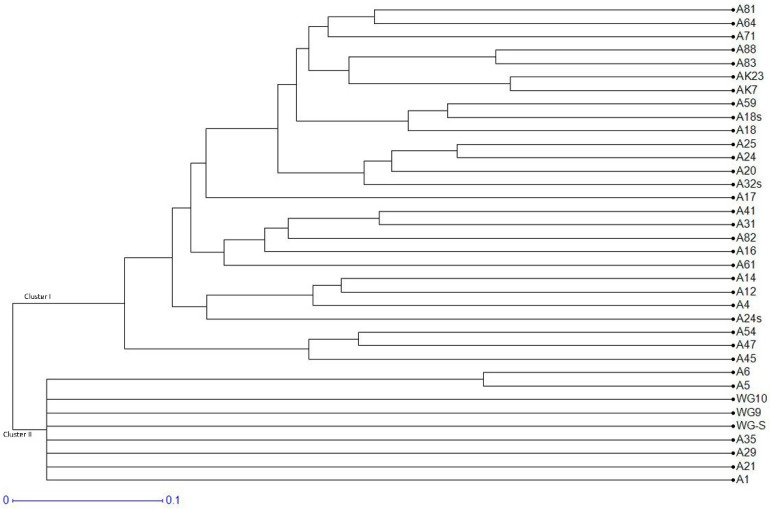
UPGMA dendrogram from SSR data of 36 isolates of *Bipolaris sorokiniana* amplified by 55 microsatellites.

**Figure 4:**
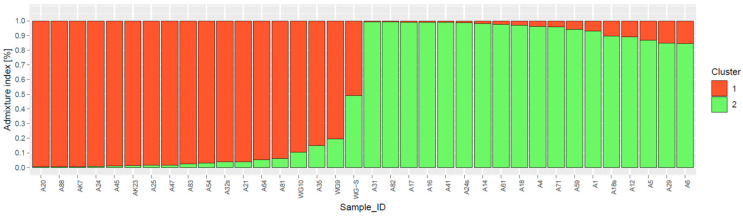
Bayesian-model-based estimation of population structure (*K* = 2) for the 36 *Bipolaris sorokiniana* isolates in 2 pre-determined populations. Each group is separated by a black vertical line. Numbers in the *y*-axis show admixture index (%).

The UPGMA-based dendrogram showed spatial clustering and separated the 36 isolates of *B. sorokiniana* into two different groups (Figure 3). Cluster I consisted of twenty-seven isolates (A81, A64, A71, A88, A83, AK23, AK7, A59, A18s, A18, A25, A24, A20, A32s, A17, A41, A31, A82, A16, A61, A14, A12, A4, A24s, A54, A47, A45), and the majority were collected from Indo-Gangetic plain regions (IG Plain regions) representing Punjab, Haryana, Uttar Pradesh, and Bihar states. Cluster II includes nine isolates (WG-S, WG9, A35, A29, A6, A5, WG10, A21, and A1), mostly representing Hill regions, and were collected from Uttarakhand and Himachal Pradesh states. Intermixing of few isolates was also observed. There were several sub-groups in both clads, demonstrating genetic diversity within and between isolates from both zones. Excluding loci with null alleles, population structure analysis using STRUCTURE 2.3.4 (Pritchard et al., 2000) revealed a strong signal with a single obvious peak at *K* = 2 for the genetic association among *Bipolaris sorokiniana* isolates (Figure 4).

## 4. Discussion

During standard phytopathological practices of pathogen isolation from symptomatic leaf tissues, researchers obtain multiple microbes on their culture media but generally they discard the disinterested microorganism and only focus on their key interested pathogen using purification techniques, leading to loss of some valuable information, and possibly there is actually a vital role of the discarded microflora on the disease epidemic. A growing understanding of plant pathogen diversity and prevalence has revealed that many diseases formerly assumed to be caused by a single primary agent are actually the consequence of complex interactions between many taxa and the host. Even when a primary agent is recognised, its action is frequently mediated by additional symbionts. As a result, the paradigm of one pathogen–one disease is giving way to the pathobiome concept [36]. The result shows that a mixed culture of fungi were obtained from leaf blight samples and they were morphologically identified as *Bipolaris* spp., *Curvularia* spp., and *Alternaria* spp. The most predominant fungus observed was of *Bipolaris sorokiniana*. Similar multipathogenic fungal complex association was observed in the case of blight disease of maize [13,37]. In this study, leaf blight of wheat is also caused by a complex of fungal pathogens, but *Bipolaris sorokiniana* is the predominant causal agent in India and has also been reported to cause substantial yield abatement in warm humid South Asia (e.g., India, Nepal, and Bangladesh) and other major wheat-growing countries such as Canada, the United States, Brazil, and Australia [38]; similarly there was a first report of *Curvularia inaequalis* and *Bipolaris spicifera* causing leaf blight of Buffalograss in Nebraska [39]. The present study focused on leaf blight complex keypathogen *B. sorokiniana* isolates and its SSR marker development, genetic diversity, and population structure study in Indian geographical settings. The number of population genetic studies on pathogenic fungus has expanded as a result of this work. Comparatively few population genetic studies of plant pathogenic fungus have been conducted thus far [40,41]. Understanding pathogen genetic diversity and population structure at the spatial scale is necessary to comprehend how pathogen populations can proliferate, become more aggressive, evolve fungicide resistance, and surpass host resistance [42,43].

In this study, 55 polymorphic markers were developed and applied to assess the genetic diversity of *Bipolaris sorokiniana* isolates. The markers found a variety of polymorphisms, from very informative to almost informative. A marker’s PIC value measures a locus’s ability to discriminate between different genotypes while taking into consideration the number and relative frequency of alleles.

The outcomes of AMOVA confirmed the existence of genetic diversity in the *B. sorokiniana* population in India. Within populations of *B. sorokiniana* isolates, variation varied to the maximum extent (90%). The Indian *B. sorokiniana* populations did exhibit some gene diversity, although it was not very high. According to the high migration rate (N_M_-3.261) estimations, the comparatively low F_ST_ value (0.071) between the *B. sorokiniana* population analysed in this study suggested little differentiation across the groups that may be attributable to gene flow among regions; therefore, in the context of the current study, migration is more significant than genetic drift. It is also a well-established fact that seedlings or young plants contract an infection at the roots, crown, or other below-ground locations, and that the infection later spreads to the above-ground parts. Conidia then form, and secondary conidial spread occurs with the aid of wind, sprinkling rain, or human interventions. Furthermore, it is possible that environmental factors, geographic location, and wheat cultivar genotypes may have an impact on the genetic variations in *B. sorokiniana* [7,8].

Understanding of the pathogen’s biology, evolution, and potentially adaptive genotypic diversity in the species is improved by information on the population structure of Bipolaris populations from various territories [14]. The substantial genetic similarity among populations indicates that the *B. sorokiniana* isolates from the Indogangetic plains (IGPs) regions of India are closely related. In addition, the population of isolates were divided into the Hill Regions (HR) and Plain Regions (PR) subpopulations on the basis of population genetic analysis. The unweighted neighbour-joining technique and STRUCTURE analysis all supported and showed evidence of mixing between the two populations. The two distinct sub-groups within *B. sorokiniana* isolates from Plains and the low level of genetic differentiation they exhibited were the results of an intense occurrence of genetic discrimination that presumably occurred at a low level due to migration. The findings are consistent with other studies by [42,43,44] (Hamelin et al. (1995), Braithwaite et al. (2004), and Zhou et al. (2008)), which found that basidiomycetes fungi have a limited degree of genetic variation. Furthermore, it appears that *B. sorokiniana* isolates probably moved from plain regions to parts of the hilly regions through anthropogenic activities associated with the production and distribution of wheat seed. The limited and sympatric distribution of two discrete clusters (Hills and Plains) in the wheat sampling areas and the fact that two different wheat-growing terrains were clustered in the same lineages were strong arguments in favour of this assertion. Therefore, the plain indogangetic regions population was most likely migrated into the hill regions of Uttrakhand and Himachal Pradesh in recent times. It is not supported by evidence of admixture between isolates from the various locations to divide isolates into clearly distinct subpopulations. The PhiPT value (0.072) among the *B. sorokiniana* populations examined in this study showed moderate differentiation among the groupings, which may be related to gene flow between locations. The moderate level of variability in populations of *B. sorokiniana* seen in this study might be explained by the long-distance conidial dispersal that might facilitate pathogen dissemination in wheat-growing regions of India. This dissemination could be connected to the trade of wheat germplasm between farmers, the seed business, and scientists.

## 5. Conclusions

The SSR markers developed in this study were employed to analyse the genetic diversity and population structure of *B. sorokiniana* isolates from India’s wheat-growing regions. Despite geographical differences, population-observed genetic diversity was lower than predicted, pointing to regional planting material trades and inoculum distribution between the regions. This research produced data that can be used to better understand the biology of the pathogen and its evolutionary potential, as well as to lay the groundwork for future research on disease development, host–pathogen interactions, and the creation and application of disease-resistant wheat varieties. Additionally, the knowledge gained from this study can be used to create new, precise primers for the identification and detection of *B. sorokiniana* from the wheat leaf blight complex. The number of population genetic studies on pathogenic fungi will be increased as a result of this research. Comparatively few population genetic studies of pathogenic fungus have been conducted thus far [45]. Understanding pathogen genetic diversity and population structure at the spatial scale is necessary to comprehend how pathogen populations can proliferate, become more aggressive, acquire fungicide resistance, and surpass host resistance [46,47,48,49].

## Figures and Tables

**Table 1:** Characteristics of 55 polymorphic microsatellite markers developed in this study for population genetic diversity analysis of *Bipolaris sorokiniana* isolates.

Locus	Forward Primer Sequence (5′-3′)	Reverse Primer Sequence (5′-3′)	Tm (°C)	Allele Size (bp)	Motif	Repeat
BS-1	GGAGATGTGTGTCATGGTGC	GGTCTTGATATTCCGTGTGCG	58	265	AGT	44
BS-2	ATGTCACTTCCGACTCCAGC	TTTTGCTTCGGTTGCTTCGG	59	224	ACA	42
BS-3	TGAGATGGTTGCAAAGGGGG	TCAACTCCATATTGCTTGGACC	60	243	AGA	34
BS-4	GTTCTTGTTCTGCAGGTGCG	GAACAGCAGAGAAAAGGCGC	60	214	ACT	30
BS-5	CACAGATGCTTTATCGCGCG	GCAACTGAAAACGGCAAATCC	60	183	GTA	24
BS-6	GATTTTGATCGAAGGGGCGG	ACCTCATATGCGCACAAAAGG	59	157	TTC	24
BS-7	TGGATTTGTCGGAGTTGAATTGC	TTTCCAACGGAAATTCGCGG	60	199	CTT	21
BS-8	TGAGGATGAGGTTGTTGCGG	AACATACGCCCACCTCATCC	60	174	GAG	21
BS-9	CGGTTAGCCACAGCAAAGC	TGTATTGTTCAAGCTGGCGC	59	153	TAG	20
BS-10	CAACATGCTCGTTACCGTGG	TCACGCATCTAAGCAGCAGC	59	125	TAC	20
BS-11	GGAACCTACTCCGACGTTGC	ATGTACAGACGCACGTCAGC	60	209	TGA	18
BS-12	ACGGGTAAATCATCGGTGCC	TGGTGCAGGTATGAAGACGG	60	175	ACT	18
BS-13	TTGCTGCTGCCTTGTATTGC	GCGTGCTGCAACAATGGG	59	130	ACT	18
BS-14	ACGAGTCCTTTTTACCACAGC	ATCTGGCGTACTTTCCGTCC	58	177	GAA	17
BS-15	GACACACTCGACTCGATGCC	CGCGAGGTTACTGGGATTGG	60	135	ACA	17
BS-16	AGATTATCAGGCCTCCACAGG	CTCTCCAGGCACCAACCG	58	191	ATC	16
BS-17	ACACTCGCCTTTAGTTTGGC	TGGTATGTCGTCCCAAAGCC	58	180	TTC	16
BS-18	TCCACCCCAATTCTATACTTACTACC	TAATCAGAGGGGCAAAGGGC	59	209	TAG	15
BS-19	ACCGTCCTACCCAGATACCC	TTGAGGATGGGGTGGGATCC	60	184	AGA	15
BS-20	TTGCCCATTGCTCGTTACCC	GAGGGGTTTCAGCAGTAGGG	60	183	CTA	14
BS-21	AGGCTGAAGCTGACAAAGGC	TTGGAGGAGAAGGAGGACGG	60	182	GAA	14
BS-22	CGAGCACACAGTCGTCTAGG	TTGTTCGTTTTGCGTGTCGG	60	182	TCA	14
BS-23	AGGCATTCAGTCCGTTAGTCC	CTTATTGCCGGCTGCTTACG	59	125	GTCA	16
BS-24	ATGTGGGAATACGGGGAAGG	TTCAGCCAAGTCTCTTGTGC	59	205	AGAA	14
BS-25	AGACCATCTGTTGCCCAACC	CAGACTGATTCCTTGTCGAGC	60	165	ATAC	14
BS-26	GCGTTTGCTTTCGATCGTCC	AGGCTGGAGAGGAGAGTTGG	60	157	AAGA	12
BS-27	AGACATTGAGGCAGTCGTGG	GGAAAACAGGCCGTTGTTGC	60	148	GGCT	12
BS-28	GACATCGTATCTGCCGTGGG	AAAGCTGTCAAATTGCGGGC	60	174	TACA	11
BS-29	TCAAATGCAATGTATTCTCTACCCG	CACGTCCCATAACGGATTGC	59	159	TCTT	11
BS-30	ACAACCTGCCACTATCACGG	CCTAGTGGATGGGCAATGGG	60	169	CCAT	10
BS-31	TGCATCACTGTAAGCCCTGG	TCCCAGCTTCAATGCCTTGG	60	167	CATA	10
BS-32	TTTTCTTTCTCTCCGCACGC	GTCTTGGGGGTGGACAAGG	59	168	AGAA	9
BS-33	TTTTGATCGAGGTCTAACAGGC	GCTCAATCGAGGAACTATGCC	58	168	CTTT	9
BS-34	GAATAGGGAGTGGACGAGAGC	ACAAACGCTGCGTAGATTTCC	59	196	AAAG	8
BS-35	GATTGGGCCAGTTGAAACCG	TGCCACCCTCCTCTACTACC	59	188	GGTT	8
BS-36	CTGGTAGCGGTAGTGGTAGC	CTTGTAGAGAGGAGCCCTGC	59	158	GTGTA	16
BS-37	CGTTCATTTTCTCCGCCAGG	TGGCATATGAATCCTGGGGG	59	165	AAAAG	12
BS-38	CATCAGCCAAACCGTTGACG	TGTACTCTACACGGATGCATACG	60	176	ACTAC	9
BS-39	GCCCCTAGATGAGAACTCGC	GCGAAATTTGCTGCAATCCC	59	178	TCGTG	8
BS-40	TCAGTATCTAGTGCGCACCC	TGTGCATTGTTGTGCTGTCC	59	171	TGCCC	8
BS-41	CTTCCATACTAGTCGGTCCCC	GGCAGGGCTTTCTTTTTGGG	59	178	CACTA	7
BS-42	GACTAGTACTGGGCGATGGC	ACCAATCCTACTCGGCATGC	59	204	CGCAA	6
BS-43	CTGCCCTAGAGTAAGACGGC	TGGAGTGTGTTGCTGCTAGG	59	166	CATCC	6
BS-44	CCTACCTCCTCCCACTACCC	AAGTGATAGTGCGGGTGTGG	60	160	CCATC	6
BS-45	CCGTTCACATGCCGTAAACG	CTGGGCGTGGTGTTTGTCG	60	151	CCATC	6
BS-46	CTTTGCATGTTCCTGACCCG	CTTGCAACTCCAACATGGCC	59	259	AGCAGT	17
BS-47	TCGTCTACGCCACGAATAGC	CCTCTAATGCGACGCGACG	59	249	TAGATG	11
BS-48	TCTAGGCGTAGAGTGCTCCC	CCTGTCGAGCTGAAAATGCG	60	159	GCTCAT	10
BS-49	TTTCGCTGAAACTTGCTCGC	TGAAGCCGAAGATGAGCAGG	60	121	TCCTGT	8
BS-50	GTATGGGGCAGACTGGTAGC	CTCGTCCACGTCTACATCCC	59	199	TGCTGT	7
BS-51	CAAATGTCCGGCGATGTTGG	GCTAACATGCAGCCAACACC	60	192	TGATTC	7
BS-52	TAGGACTTGTTGCGGCTAGG	ACATGCTACACGGACACACC	59	179	ACCAGC	6
BS-53	TCCTTGTCCTTGTCCTTGTCC	AGCCCTATGGTCACGAATGC	59	179	TTCTCT	6
BS-54	GGGCTGGACGAGTGATATGG	TGCTGATACCGTTGCTGTCG	59	160	AGGAGC	6
BS-55	ATCTTTTCGTGCAGGGGAGG	TCGATCCTCAAATAGCGCCC	60	158	TGTGGC	6

**Table 2:** Polymorphism analysis and diversity indices of the microsatellite loci used in the study.

Marker	Major Allele Frquency	Allele No.	Availability	Gene Diversity	Observed Heterozygosity	Expected Heterozygosity	Polymorphic Information Content	Shannon’s Information Index
BS-2	0.5238	3.0000	0.5833	0.5397	0.4472	0.5528	0.4368	0.8468
BS-3	0.8333	2.0000	0.5000	0.2778	0.3444	0.3556	0.2392	0.5297
BS-4	0.5000	3.0000	0.7222	0.5355	0.4546	0.5461	0.4276	0.8287
BS-5	0.8333	3.0000	0.5000	0.2901	0.2016	0.2984	0.2686	0.5566
BS-6	0.5238	2.0000	0.5833	0.4989	0.4892	0.5111	0.3744	0.6921
BS-7	0.5714	3.0000	0.5833	0.5261	0.4611	0.5389	0.4292	0.8324
BS-8	0.8947	2.0000	0.5278	0.1884	0.5065	0.4935	0.1706	0.3365
BS-9	0.8571	2.0000	0.5833	0.2449	0.5491	0.5509	0.2149	0.4101
BS-10	0.7391	3.0000	0.6389	0.4197	0.6851	0.6149	0.3819	0.7356
BS-11	0.7500	3.0000	0.4444	0.3984	0.5242	0.4758	0.3542	0.7775
BS-13	0.7143	2.0000	0.1944	0.4082	0.5604	0.4396	0.3249	0.5983
BS-14	0.8750	2.0000	0.4444	0.2188	0.1742	0.1258	0.1948	0.3768
BS-15	0.8462	2.0000	0.3611	0.2604	0.4292	0.5708	0.2265	0.4293
BS-16	0.8182	2.0000	0.3056	0.2975	0.5883	0.4117	0.2533	0.4741
BS-17	0.5000	2.0000	0.5556	0.5000	0.4872	0.5128	0.3750	0.6931
BS-18	0.8750	2.0000	0.2222	0.2188	0.4667	0.5333	0.1948	0.3768
BS-19	0.6667	2.0000	0.4167	0.4444	0.5402	0.4598	0.3457	0.6365
BS-20	0.6364	3.0000	0.3056	0.5124	0.4632	0.5368	0.4442	0.8633
BS-21	0.5400	3.0000	0.6944	0.5864	0.4016	0.5984	0.5112	0.9726
BS-22	0.6957	3.0000	0.6389	0.4612	0.5285	0.4715	0.4075	0.7966
BS-23	0.3947	1.0000	0.2278	0.3942	0.2431	0.2131	0.3674	0.4895
BS-24	0.5000	5.0000	0.8056	0.6474	0.4412	0.5688	0.5932	1.2415
BS-25	0.5000	3.0000	0.7222	0.5888	0.4997	0.5003	0.5042	0.9632
BS-26	0.5000	3.0000	0.4444	0.6172	0.3629	0.4371	0.5439	1.0239
BS-27	0.9167	2.0000	0.6667	0.1528	0.8441	0.8563	0.1411	0.2868
BS-28	0.5238	3.0000	0.5833	0.6122	0.5728	0.6272	0.5436	1.0221
BS-30	0.6111	4.0000	0.5000	0.5432	0.4413	0.5287	0.4800	0.9779
BS-31	0.5500	2.0000	0.8333	0.4950	0.4966	0.5034	0.3725	0.6881
BS-32	0.8846	2.0000	0.7222	0.2041	0.7919	0.8081	0.1833	0.3576
BS-33	0.7532	2.0000	0.8332	0.1932	0.3212	0.2155	0.3211	0.6236
BS-34	0.7420	2.0000	0.5278	0.2716	0.3321	0.4120	0.3982	0.4896
BS-35	0.5200	3.0000	0.6944	0.5344	0.4547	0.5453	0.4282	0.8366
BS-36	0.8333	2.0000	0.6667	0.2778	0.7163	0.7837	0.2392	0.4506
BS-37	0.8261	2.0000	0.6389	0.2873	0.5131	0.4871	0.4461	0.6693
BS-38	0.6522	3.0000	0.6389	0.4991	0.4899	0.5101	0.4337	0.8417
BS-39	0.9167	2.0000	0.3333	0.1528	0.8406	0.7594	0.1411	0.2868
BS-40	0.8333	4.0000	0.8333	0.2933	0.7017	0.7983	0.2763	0.6089
BS-41	0.4228	2.0000	0.4722	0.2974	0.3211	0.4810	0.3902	0.5608
BS-42	0.8261	2.0000	0.6389	0.2873	0.7063	0.2937	0.2461	0.4623
BS-43	0.4737	3.0000	0.5278	0.5873	0.4969	0.5031	0.4988	0.9551
BS-44	0.5037	2.0000	0.4111	0.5109	0.4211	0.4140	0.4012	0.6221
BS-45	0.6667	2.0000	0.6667	0.4444	0.5461	0.4539	0.3457	0.6365
BS-46	0.5296	2.0000	0.5278	0.4924	0.2291	0.2312	0.3122	0.4287
BS-47	0.5714	3.0000	0.5833	0.5805	0.4053	0.4947	0.5157	0.9773
BS-48	0.6957	3.0000	0.6389	0.4461	0.5441	0.4561	0.3782	0.7393
BS-49	0.9231	2.0000	0.3611	0.1420	0.8523	0.8477	0.1319	0.2712
BS-50	0.7218	2.0000	0.3611	0.5107	0.2121	0.2311	0.2101	0.3214

## Data Availability

Not applicable.

## Supplementary Materials

The following supporting information can be downloaded at https://www.mdpi.com/article/10.3390/jof9020153/s1, Figure S1: Representative gel pics showing polymorphism of SSR markers; Table S1: Collection of wheat leaf blight samples from different Agro-climatic regions of India.
