# Supplementary material for: Development of Diagnostic Markers and Applied for Genetic Diversity Study and Population Structure of Bipolaris sorokiniana Associated with Leaf Blight Complex of Wheat
Source: J Fungi (Basel). 2023 Jan 23;9(2):153. doi: 10.3390/jof9020153 (PMC9968152; doi:10.3390/jof9020153)
Supplement: Supplementary file 1 [file jof-09-00153-s001.zip › jof-1980944-supplementary.pdf]

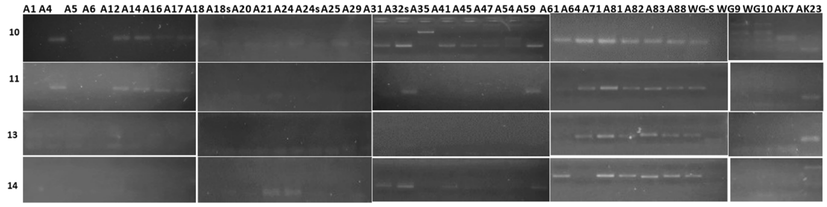

a

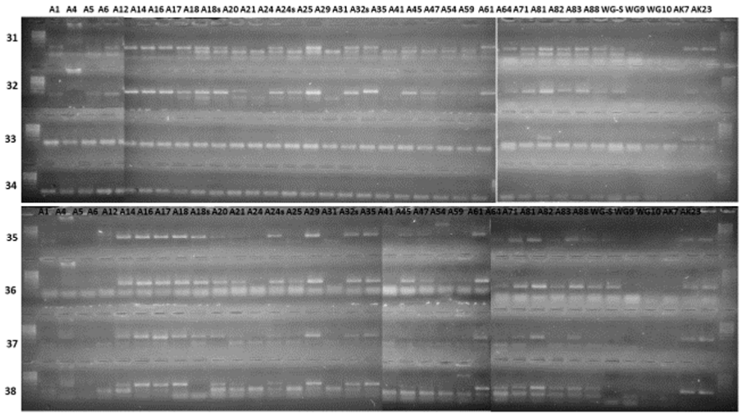

b

Supplementary Figure S1.- Representative gel pics showing polymorphism of SSR markers

Supplementary Table S1. Collection of wheat leaf blight samples from different Agro-climatic regions of India.

Agro-climatic region	Place of collection	Geographical Coordinates		Location	Source of isolation	Host	No. of samples	Annual pluviometry (mm)	Average temperature (Kharif)	Cultivars of wheat	Year of collection
		Latitude	Longitude								
Trans-Gangetic Plains Region Zone -6	Jalandhar ,Punjab	31.19N	75.38E	India	Diseased leaf samples	Wheat	62	750–1250	7°C to 28°C	HD 3226, Karan Vandana, WH1080, Pusa Ujala, PBW- 343	2017-18
	Ludhiana ,Punjab	30.98N	75.72E	India	Diseased leaf samples	Wheat					
	Khokhar Kalan ,Punjab	30.01N	75.37E	India	Diseased leaf samples	Wheat					
	Jajjal, Haryana	28.96N	77.18E	India	Diseased leaf samples	Wheat					
	Ludhiana, Punjab	30.98N	75.72E	India	Diseased leaf samples	Wheat					
	Jalandhar , Punjab	31.19N	75.38E	India	Diseased leaf samples	Wheat					
	Ferozepur, Punjab	30.96N	74.65E	India	Diseased leaf samples	Wheat					

	Gurdaspur, Punjab	32.06N	75.47E	India	Diseased leaf samples	Wheat					
	Madoke, Punjab	31.69N	74.69E	India	Diseased leaf samples	Wheat					
	Dhilwan, Jalandhar, Punjab	31.32N	75.65E	India	Diseased leaf samples	Wheat					
	Sangrur, Punjab	30.25N	75.77E	India	Diseased leaf samples	Wheat					
	Faridkot, Punjab	30.63N	74.88E	India	Diseased leaf samples	Wheat					
Upper Gangetic Plains Region Zone -5	Mahmood Pura Lakarsandha, Uttar Pradesh	29.49N	77.66E	India	Diseased leaf samples	Wheat	35	750-1500	7°- 30°C	Pusa Malawi HD 3171, *UP-2338,* PBW-343, PBW – 373, UP -2425, Ujiar. Gangotri, Naina	2017-18
	Katka, Uttar Pradesh	28.05N	80.16E	India	Diseased leaf samples	Wheat					
	Kadrauli Banger, Uttar Pradesh	27.07N	79.89E		Diseased leaf samples	Wheat					
	Kanpur Dehat, Uttar Pradesh	26.47N	80.11E	India	Diseased leaf samples	Wheat	48				
	Arrapahadpur, Uttar Pradesh	27.41N	79.56E	India	Diseased leaf samples	Wheat					

	Ghosipura, Uttar Pradesh	29.99N	77.49E	India	Diseased leaf samples	Wheat					
Middle Gangetic Plains Region	Katihar ,Bihar	25.51N	87.59E	India	Diseased leaf samples	Wheat		1000-2000	9°C-30°C	Ganga (HD 2643), PBW-443, DBW-14, HD-2824, HUW 468	2017-18 and 2020-2021
	Mau ,uttar pradesh	25.53N	83.29E	India	Diseased leaf samples	Wheat					
	Sheohar, Bihar	26.52N	85.30E	India	Diseased leaf samples	Wheat					
	Munger, Bihar	25.38N	85.92E	India	Diseased leaf samples	Wheat	5				
	Naraenpur Sarai, Bihar	25.26N	87.33E	India	Diseased leaf samples	Wheat					
	Taiwara, Uttar Pradesh	25.44N	81.65E	India	Diseased leaf samples	Wheat	23				
	Mirzapur, Uttar Pradesh	27.57N	80.61E	India	Diseased leaf samples	Wheat					
	Ghazipur, Uttar Pradesh	25.71N	83.50E	India	Diseased leaf samples	Wheat					
